# Abstract of Proceedings of the Buffalo Medical Association

**Published:** 1869-07

**Authors:** Wm. C. Taylor

**Affiliations:** Sec’y pro tem.


ART. II.—*Abstract of Proceedings of the Buffalo Medical Association.*

TUESDAY EVENING, July 6, 1869.

Meeting called to order by Vice President Wetmore. Members present—Drs. Wetmore, Miner, White, Potter, Diehl, Abbott, Burger and Taylor.

Moved by Dr. White, and carried, that the report of the Treasurer be accepted; that each member be furnished with a statement of his account, and that the representation of each member as to payments made, etc., be received.

DR. MINER presented a photograph which he had received from Dr. D. Ingraham of Kennedy, N. Y., of a man some thirty years old, supposed to be suffering from elephantiasis. The history of the case is as follows: Some ten years since, the patient's previous health having been good, the left leg began to increase, and has continued to increase in size with but one intermission, which occurred some eight or nine years ago, when the man was under the care of Dr. Frank H. Hamilton. The treatment then pursued seems to have been of benefit, but the man left Buffalo before a cure was effected. Since this time he has been gradually growing worse, and the leg now measures, near the body, 2 feet 4¾ inches, around middle of thigh 4 feet 3 inches, just above the knee 2 feet 1 inch, and below it 2 feet 9 inches. Formerly he weighed some 140 lbs.; his weight is now over 300 lbs. The upper portion of the tumor pits on pressure, while the lower portion appears scaly. Its surface is rendered irregular by sulci and from the most dependent posterior part of the greatest enlargement a fetid discharge escapes. The man is a lumberman by trade, and his habits have been rather irregular. Has been exposed to hardships and drank freely. There is no syphilitic history. During these ten years the general health has been fair, with the exception of an attack of typhoid fever, and usually he has pursued his vocation in the spring, going down the river on rafts.

Dr. Miner then remarked that he did not know what the treatment, which had proved beneficial, had been, but thought bandaging, by controlling the circulation and promoting absorption, might have been of service. As to present treatment, he knew of none unless a surgeon could be found willing to perform amputation or ligation of the artery. He doubted if success would attend either operation.

Dr. Wetmore mentioned that elephantiasis was oftener seen and better known in warm climates than in our own; that the disease was very apt to attack the scrotum, and spoke of a case reported by Dr. Sunn, where the tumor was so large that the patient was obliged to wheel it in a barrow before him when he walked.

Reverting to prevailing diseases, Dr. White said that whooping-cough was prevalent; this he had often noticed to be the case when many were suffering from scarlet fever and measles.

Dr. Miner then suggested that Dr. White be invited to give his views upon the treatment of whooping-cough.

Dr. White said he believed pertussis to be a self-limited disease, and that much, if not all, depended upon hygienic treatment. That it was a great mistake to confine patients suffering from this disease, to a hot and impure atmosphere, as it rendered them particularly liable to complications. That our duty consisted in conducting our patient safely through the disease, and not by endeavoring to cut it short by severe medication. The invigorating treatment was by far the most efficacious; pure air and plenty of it, out of doors, with good food. Many medicines had been recommended, among them iodide of potassium, nitrate of potassa, cochineal, etc. Of late bromide of ammonium and bromide of potassium had been used seemingly with success. Chloroform with bromide of ammonium sometimes relieved the spasm, and counter-irritants were indicated under certain circumstances. Whooping-cough he considered to be but little under the control of medicines, but recommended attention to be paid to hygienic measures, as these were our best agents in this disease. Ordinarily too much treatment was resorted to. Emetics, sudorifics and anodynes were formerly given *ad libitum.* This mode of treatment the doctor entirely disapproved.

Dr. Miner said, he was glad to obtain for the profession the results of Prof. White's extensive experience and observation in the treatment of this common disease of childhood, since he believed the custom was far too common to trust to medicine, or at least, to prescribe medicine, rather than trust to nature and time. He held whooping-cough to be beyond the control of medicinal agents, and said that the effects of time with proper care were not duly considered. He knew of no medicine which would control or limit the disease, and believed bromide of potassium to be of little, if any use, and sometimes even injurious. Anodynes by procuring sleep might in some cases be of service. He believed it generally best to leave the disease to time and nature, unless, as Prof. White had remarked, complications with other diseases made medication proper.

Dr. Wetmore stated that his mode of treatment was to palliate the symptoms during the earlier stages, when he generally exhibited some soothing expectorant. Later he gave alcoholic stimulants, of which he preferred rum given with molasses; he had also procured good results with belladonna combined with bromide of potassium.

Dr. Abbott read the following note, which he intends sending to the members of the profession, and submitted the same to the Society:

Buffalo, July 5, 1869.

*To the Members of the Erie County Medical Society:*

Dear Sirs:—Having spent most of the time for the past year in New York, studying ophthalmology and otology under the instruction of Drs. Agnew, Noyes, Roosa, etc., at the New York Eye and Ear Infirmary, and the Brooklyn Eye and Ear Hospital, I have resumed practice in Buffalo, intending to devote special attention to diseases of the eye and ear.

I am aware that a prejudice against specialists exists in the minds of many of my professional brethren, but soon after I commenced the study of this branch of medicine, I found that it was so extensive that it would be impossible to obtain a thorough knowledge of it and continue in general practice, and I hope to gain your countenance and favor in thus informing you of my intention to practice only in this department of medicine, and trust that I may be sustained by you in attempting to rescue the public from the hands of advertising quacks.

Very respectfully, FRANK W. ABBOTT, M. D.

*References*—Thomas F. Rochester, M. D., Buffalo.
James P. White, M. D., "
Julius F. Miner, M. D., "
Charles E. Rider, M. D., Rochester.

DR. WHITE said that much had been done by specialists for the progress of medicine; that the great draw-back to devoting the entire attention to one subject was the lack of interest it engendered in general medical knowledge, and that it was necessary to be well grounded in all medical sciences in order to be a successful specialist. He saw no harm in having the note forwarded to the members of the Erie County Medical Society, particularly as Dr. Abbott, whom he knew personally, was a good general practitioner. He also remarked that if any member have objection to such proceeding on the part of Dr. Abbott, that he now state such to be the case before the Society.

DR. MINER considered the notice entirely unobjectionable, and looked upon it not as an advertisement, but simply as a notification to the profession that Dr. Abbott intends to devote his attention particularly to the eye and ear. He also said that he did not think that general practitioners were sufficiently well acquainted with many of the more intricate diseases of the eye to treat them with success, unless they made them a special study. If any physician did truly study with earnestness and care diseases of any class, and perfect himself in a complete understanding of them, his attainments would certainly be known and appreciated by a liberal profession. For himself, he would give to all such a cordial fellowship.

Adjourned. WM. C. TAYLOR, *Sec'y pro tem.*

